# Disability and Patient-Reported Satisfaction in Women with Idiopathic Intracranial Hypertension: A Comparative Study of Venous Sinus Stenting and Medical Management

**DOI:** 10.3390/diagnostics14222572

**Published:** 2024-11-15

**Authors:** Ortal Buhbut, Hadas Ben Assayag, Sapir Aharoni-Bar, Maor Epstein, Erez Tsumi, Tamir Regev, Anna Bunin, Asaf Honig, Bar O. Kotaro, Gal Ben Arie, Anat Horev

**Affiliations:** 1 Department of Ophthalmology, Soroka University Medical Center, Ben-Gurion University of the Negev, Beer Sheva 8453227, Israel; ortalbu17@gmail.com (O.B.); erezts@clalit.org.il (E.T.); mr.tregev@gmail.com (T.R.); annabunin@hotmail.com (A.B.); 2 Faculty of Medicine, Tel Aviv University, Tel Aviv 69978, Israel; hadasben97@gmail.com; 3 Ben-Gurion Medical School, Soroka University Medical Center, Beer Sheva 8453227, Israel; 4 Faculty of Health Sciences, Ben-Gurion University of the Negev, Beer Sheva 8410501, Israel; maorep@post.bgu.ac.il; 5 Department of Neurology, Soroka University Medical Center, Ben-Gurion University of the Negev, Beer Sheva 8453227, Israel; asaf.honig2@gmail.com; 6 Medical School, Faculty of Health, Palacky University, 77900 Olomouc, Czech Republic; bar.kotaro@gmail.com; 7 Department of Diagnostic Imaging, Soroka University Medical Center, Ben-Gurion University of the Negev, Beer Sheva 8410501, Israel; gal_b_a@yahoo.com

**Keywords:** carbonic anhydrase inhibitors, headache, idiopathic intracranial hypertension, transverse sinus stenting, quality of life, questionnaire

## Abstract

Objective: Patients with chronic idiopathic intracranial hypertension (IIH) commonly experience a high level of disability and low satisfaction with medical treatment. We aim to evaluate long-term functional improvement and patient satisfaction in IIH patients with similar symptoms by comparing venous sinus stenting (VSS) to standard medical therapy. Methods: We conducted a cross-sectional questionnaire study of 111 IIH patients, comparing 37 adult female patients who underwent venous sinus stenting with 74 patients treated medically. Propensity score matching was used to balance age and presence of papilledema at presentation between groups. Headache-related disability was evaluated using the Migraine Disability Assessment Scale (MIDAS), while general function and treatment satisfaction were assessed using custom questionnaires. Electronic medical records and the results of imaging upon diagnosis were reviewed retrospectively. Results: The stented group reported significantly better outcomes in physical well-being (median 4.0 vs. 1.0, *p* < 0.001), task completion (4.0 vs. 1.0, *p* < 0.001), work/school persistence (5.0 vs. 1.0, *p* < 0.001), and mental well-being (4.0 vs. 1.0, *p* < 0.001). Additionally, the stented group had a lower proportion of patients with severe MIDAS (MIDAS > 4, 24.3% vs. 47.9%, *p* = 0.017). Logistic regression suggested venous stenting as a protective factor against severe MIDAS scores (OR = 0.174, *p* = 0.004). Conclusion: Cerebral venous stenting in patients with IIH is associated with lower disability and higher patient satisfaction from medical treatment compared to those treated with medications only. These findings suggest that venous sinus stenting may be a valuable treatment option for selected IIH patients. However, larger prospective studies are needed to further validate our results.

## 1. Introduction

Idiopathic intracranial hypertension (IIH) is a neurological disorder characterized by elevated intracranial pressure (ICP) without an identifiable cause. Symptoms typically include chronic headaches, visual disturbances, and pulsatile tinnitus (PT) [1]. Beyond these symptoms, IIH is also associated with significant impairment in daily activities, increased prevalence of depression, and cognitive impairment [2,3,4]. Collectively, these factors adversely affect patients’ functional ability and diminish patient-reported treatment satisfaction [5].

While the majority of IIH patients are managed pharmacologically with acetazolamide or topiramate, these treatments have demonstrated limited efficacy in managing the full spectrum of IIH symptoms [6,7]. Consequently, only approximately 10% are referred for surgical intervention [8]. However, current pharmacological treatments have demonstrated limited efficacy in managing the full spectrum of IIH symptoms. The Idiopathic Intracranial Hypertension Treatment Trial (IIHTT) demonstrated the effectiveness of these agents in reducing papilledema; however, their impact on subjective symptoms, such as headaches and tinnitus, remains suboptimal [9]. Notably, 44% of patients continued to report headaches at 6 months post-treatment [10,11]. Moreover, recent investigations emphasize that despite papilledema resolution, significant functional deficits and quality-of-life impairments persist [2,12,13]. These findings suggest that ophthalmological resolution may not correlate with overall functional improvement.

In recent years, cerebral venous sinus stenting (VSS) has emerged as a treatment option for IIH [14,15]. This procedure aims to enhance cerebral venous drainage, as venous stenosis- has been identified as a significant factor in IIH pathophysiology [16,17,18,19]. Although data are accumulating on the impact of VSS on papilledema, visual symptoms, and other clinical manifestations, its effect on long-term patient disability and satisfaction, particularly in comparison to standard medical therapy, remains not fully understood [20,21,22]. To address this gap, our study aims to evaluate whether venous sinus stenting provides long-term functional improvements and greater patient satisfaction compared to standard medical therapy in age-matched IIH patients with similar clinical presentations.

## 2. Materials and Methods

### 2.1. Study Design and Population

This cross-sectional study evaluated disability and treatment satisfaction among female patients diagnosed with IIH, comparing those treated with standard medical therapy to those who underwent VSS. We reviewed electronic medical records of patients diagnosed with IIH between January 2012 and December 2018 at Soroka University Medical Center. To reduce potential bias due to gender differences in the pathophysiology of IIH, only female patients were included, as IIH predominantly affects females. The study population was divided into two groups based on their treatment modalities.

Medical treatment (MT) group: This group comprised female patients aged 18 years or older who were diagnosed with IIH (Benign Intracranial Hypertension ICD9: 348.2) at least six months prior to enrolment. These patients were managed solely with pharmacological treatments, specifically acetazolamide and/or topiramate, for a minimum of three months. At the time of questionnaire completion, some patients were continuing pharmacological therapy, while others had discontinued treatment following the resolution of papilledema.

Cerebral VSS group: This group included female patients aged 18 years or older who were diagnosed with IIH and underwent VSS due to inadequate response or significant intolerance to pharmacological treatment. Failure of medical therapy was defined as persistent papilledema and/or deterioration of the visual field despite optimal medical treatment. At our institution, VSS is the primary surgical intervention offered because of its low complication rates and minimally invasive nature; venrtriculo–peritoneal (V-P) shunting or optic nerve sheath fenestration (ONSF) is reserved as a second-line option. All patients in this group showed no signs of optic disc edema in their most recent ophthalmological evaluation, conducted at least six months prior to enrolment.

### 2.2. Inclusion and Exclusion Criteria

Female patients aged 18 years or older with a confirmed diagnosis of IIH, according to the revised Friedman criteria [23], were eligible for inclusion. Participants had to be at least six months post-diagnosis and have no signs of optic disc edema in their most recent ophthalmological evaluation. For the VSS group specifically, patients needed to have documented failure of pharmacological treatment or intolerance to medications as the indication for undergoing VSS. All participants had to be available and willing to consent to participate in the study.

Exclusion criteria encompassed male patients, individuals with secondary causes of intracranial hypertension, and patients lacking at least one cranial venography (either CTV or MRV) performed within the month of diagnosis archived in our radiology system. Additionally, patients without lumbar puncture opening pressure measurements at diagnosis were excluded.

Out of 210 consecutive patients diagnosed with IIH, 136 had updated contact information and agreed to participate in the study. The VSS group consisted of 39 patients who consented to participate. To enhance the validity and comparability of the study, we matched the MT group to the VSS group using propensity scores in a 2:1 ratio based on age and the presence of papilledema.

**Figure 1:**
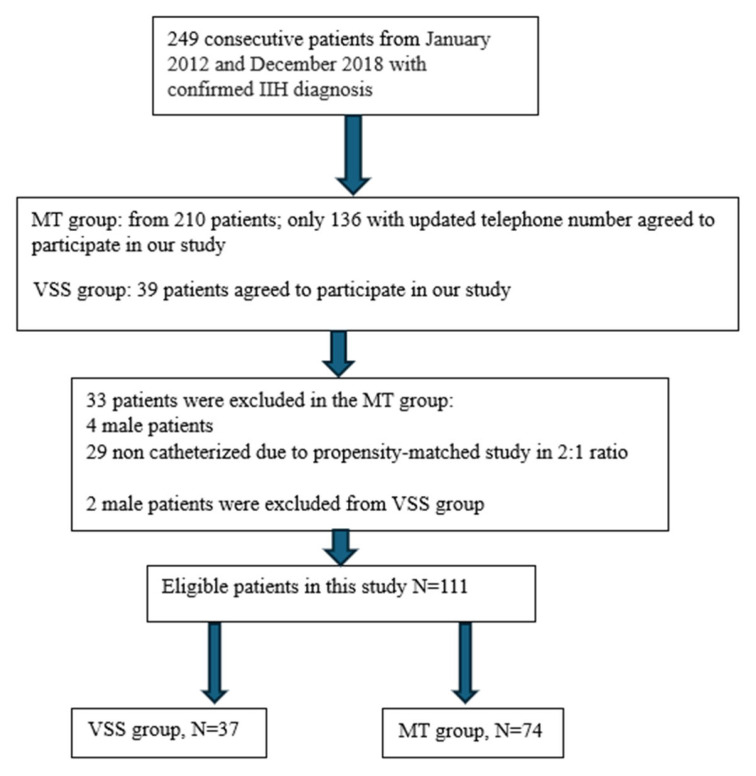
Flow diagram of patient enrollment to MT and VSS groups.

During the matching process, 64 patients were excluded. This included six male patients—two from the VSS group and four from the MT group—and twenty-nine from the MT group who did not meet the matching criteria. After completing the matching process, a total of 111 patients were enrolled in the study: 74 patients in the MT group and 37 patients in the VSS group (Figure 1).

### 2.3. Statement of Ethical Approval and Consent

This study was approved by the Institutional Review Board of SUMC (Approval No. 0278-2020). Informed consent was obtained verbally prior to participating in the study. All participants consented to the anonymized use of their clinical and questionnaire data.

### 2.4. Data Collection

Retrospective data collection was performed by four different researchers specializing in general medical, neurology, ophthalmology, and radiology. Each researcher independently extracted data from patients’ index hospitalization records and health medical organization (HMO) electronic records. To minimize bias, the researchers were blinded to each other’s data collection.

For epidemiological and clinical data, a neurologist and an internal medicine physician collected epidemiological and general medical information including age, body mass index (BMI) at the time of index hospitalization, lumbar puncture opening pressure, chronic medical conditions, and current medications.

For ophthalmological data, ophthalmological medical charts were reviewed to gather data on the presence of papilledema and visual field defects at presentation and during follow-up visits. Visual fields were tested using the Humphrey 24-2 SITA-Standard automated perimetry. An abnormal visual field was defined by the presence of suspicious findings, such as blind spot enlargement, inferonasal defect, arcuate defect, or severe visual field constriction, in conjunction with an abnormal mean deviation (MD) of less than −2 decibels (dB). For radiological data, a single radiologist analyzed imaging data to rule out structural lesions that could contribute to increased ICP. The following radiological findings supporting IIH [22,24] were evaluated:

- Empty sella turcica;
- Flattening of the posterior sclera;
- Optic nerve dural ectasia: measured bilaterally; an average optic nerve sheath diameter exceeding 5 mm was considered indicative of optic nerve sheath widening;
- Transverse sinus stenosis (TSS): assessed using the Combined Conduit Score (CCS), an index introduced by Farb et al. in 2003 [14]; each transverse sinus (left and right) was graded for patency on a scale from 0 to 4:
  - 0: 0% patency;
  - 1: Less than 25% patency;
  - 2: 25–50% patency;
  - 3: 50–75% patency;
  - 4: 75–100% patency.

The scores from both sides were summed to yield a total CCS ranging from zero to eight. A score of 8 indicates normal patency with minimal or no TSS, while a score below 5 suggests significant venous sinus stenosis. An IIH Radiological Score (IIHRS) was subsequently calculated by aggregating positive findings from the four radiological criteria listed above. The IIHRS ranged from 0 to 4, serving as an ordinal variable representing the severity of radiological features associated with IIH.

Procedural data:

Patients referred to VSS underwent an evaluation of the stenotic areas by contrast injection from the superior sagittal sinus and pressure gradient measurement pre- and post-stenosis in the transverse sigmoid sinus bilaterally. According to current criteria, VSS was performed only in patients demonstrating a pressure gradient of at least 8 mmHg across the stenotic region. A stent (Precise 7 × 40/8 × 40, Cordis, Hialeah, FL, USA) was then deployed across the stenotic area of the dominant or most severely stenotic sinus. Post-stenting, repeat gradient measurements were performed to confirm the normalization of the pressure gradient. All patients were treated with dual antiplatelet therapy, consisting of clopidogrel 75 mg and aspirin 100 mg daily. This regimen was initiated at least 5 days before the procedure and continued for 3 months post-procedure. Following the initial 3-month period, brain venography (either CTV or MRV) was performed to confirm stent patency and adequate venous drainage ipsilateral to the stent. If imaging confirmed appropriate positioning and patency, clopidogrel was discontinued, while aspirin was maintained for an additional 9 months. A second imaging study was performed before complete cessation of antiplatelet therapy.

### 2.5. Study Questionnaire

All participants were interviewed between November 2020 and October 2022 using a structured questionnaire (Supplementary Materials Questionnaire) comprising three parts as follows:

1. Demographics and medical history—the first section included 37 questions covering basic demographic characteristics, time since diagnosis, and additional medical history.

2. Migraine Disability Assessment Scale (MIDAS)—the second section featured the MIDAS, a self-report tool consisting of five questions designed to assess headache-related disability. Responses were summed to calculate the MIDAS score, classifying migraine severity into four grades: Grade I—little or no disability, Grade II—mild disability, Grade III—moderate disability, and Grade IV—severe disability.

3. Patients-reported outcome measures (PROMs)—the third section focused on PROMs and included 23 questions. Satisfaction with current medical treatment and perception of symptoms were assessed using nine questions on a Likert scale ranging from 1 (“strongly disagree”) to 5 (“strongly agree”). Side effects were evaluated with a single question rated from 0 (“not at all”) to 7 (“bothers me a lot”). The final eight questions examined functional improvement and lifestyle changes since diagnosis, consisting of yes/no responses and two additional questions on a 1–5 scale. One dichotomous question assessed the presence of PT at diagnosis and within three months post-treatment.

### 2.6. Statistical Analysis

As mentioned earlier, our cohort was matched according to age and the presence of papilledema. Matching was conducted using propensity scores, calculated via a logistic regression model that included age and papilledema as covariates. Nearest-neighbor matching was then used to match catheterized participants to those in the non-catheterized control group. Post-matching, we assessed the balance of covariates between the groups to confirm successful matching, as presented in Table 1 (mean age of 32.9 ± 10.4 in the non-catheterized group and 32.7 ± 10.0 in the catheterized group, *p* = 0.912; 85.1% papilledema in the non-catheterized group and 75.7% in the catheterized group, *p* = 0.222). Subsequent analyses were conducted on the matched sample to evaluate the effect of catheterization.

In the analyses, categorical variables were presented as counts and expressed as a percentage of their respective groups. Continuous variables were assessed for normal distribution and reported as mean along with standard deviation (SD). Ordinal variables were presented as medians and interquartile range (IQR). To compare categorical variables, we employed a chi-squared test, while continuous variables were analyzed using an independent *t*-test. A Mann–Whitney U test was used to analyze ordinal variables. All statistical analyses were considered significant at *p* < 0.05 (two-tailed) and were conducted using SPSS software (IBM SPSS Statistics, version 28).

Additionally, a binary logistic regression model was utilized to predict the probability of having severe MIDAS (Grade IV). We adjusted our model for several covariates, including catheterization intervention, age, presence of papilledema, Uramox/Topamax treatment, PT in the past 3 months, obesity (BMI ≥ 30), categorized height, and weight loss since diagnosis.

## 3. Results

A total of 111 female patients with IIH were included in this propensity-matched study, with 37 patients (33.3%) in the VSS group and 74 patients (66.7%) in the MT group. The groups were matched in a 1:2 ratio based on age and the presence of papilledema.

### 3.1. Medical History and Comorbidities

The VSS group exhibited a significantly lower lumber puncture opening pressure at diagnosis compared to the MT group (330.5 mmH_2_O vs. 377.1 mmH_2_O, *p* = 0.044). There was no statistically significant difference between the groups in time since diagnosis prior to study enrollment (*p* = 0.066). Visual field damage at diagnosis was similar between the two groups (*p* = 0.438). While BMI at diagnosis did not differ significantly, there was a trend toward higher obesity rates in the VSS group compared to the MT group (66.7% vs. 47.1%, *p* = 0.056). The analysis of comorbidities revealed significantly higher rates of polycystic ovary syndrome (PCOS) and fibromyalgia in the MT group compared to the VSS group (21.9% vs. 2.8%, *p* = 0.010, and 8.1% vs. 0.0%, *p* = 0.013). Other comorbidities, including diabetes mellitus, hypertension, hypertriglyceridemia, hypothyroidism, depression/anxiety, and post-traumatic stress disorder (PTSD), showed similar prevalence in both groups. Notably, a greater proportion of patients in the VSS group reported experiencing PT at diagnosis compared to the MT group (78.4% vs. 55.6%, *p* = 0.019).

### 3.2. Radiological Findings

Over 90% of the study cohort had significant venous stenosis, indicated by a CCS ≤ 5 at diagnosis. The presence of an empty sella turcica was significantly higher in the VSS group than in the MT group (84.6% vs. 52.4%, *p* = 0.004). However, there were no significant differences between the groups regarding optic nerve sheath dilatation and flattening of the posterior sclera. The IIHRS was not significantly different between the groups (median [IQR]: 2.0 [1.0–3.0] in the MT group vs. 2.0 [1.0–2.0] in the VSS group, *p* = 0.274), indicating overall similarity in radiological features regardless of treatment modality. Another notable outcome was the change in PT over time. In the past three months (post-treatment), the MT group had a higher prevalence of PT compared to the VSS group (50.7% vs. 16.2%, *p* < 0.001) (Table 2).

Using the MIDAS, the MT group demonstrated higher rates of disability compared to the VSS group. The MIDAS grades were significantly higher in the MT group (median 3.0) than in the VSS group (median 1.0, *p* = 0.025). A greater proportion of patients in the MT group fell into the severe disability MIDAS Grade IV compared to the VSS group (47.9% vs. 24.3%, *p* = 0.017). Specifically, MIDAS questions 1 and 2 which assess functional impairment due to headaches at work and school showed significant differences between the groups (*p* = 0.002 and *p* = 0.004, respectively). Another notable outcome was the change in the PT over time. In the past 3 months (post-treatment), the MT group had a higher prevalence of PT compared to the VSS group (50.7% vs. 16.2%, *p* < 0.001). PROMs consistently showed lower scores in the MT group compared to the VSS group. Measures of physical well-being (median 1.0 vs. 4.0, *p* < 0.001), ability to complete tasks (1.0 vs. 4.0, *p* < 0.001), persistence with work/school (1.0 vs. 5.0, *p* < 0.001), mental well-being (1.0 vs. 4.0, *p* < 0.001), and difficulty maintaining social relationships (0 vs. 1.0, *p* < 0.001) all favored the VSS group. Notably, both groups reported relatively lower scores regarding difficulty maintaining social relationships.

No significant intra-procedural and post-procedural complications were reported. Three patients developed post-procedural hematoma with no need for blood transfusions or any surgical interventions. No significant pre-stent stenosis, stent stenosis, or occlusion were reported on patients’ follow-up imaging. The VSS group reported significant improvements in headache discomfort following the procedure. The median headache discomfort score decreased from 7.0 [6.0–7.0] pre-procedure to 3.0 [0.0–4.0] post-procedure, indicating a substantial reduction in headache severity. Patients expressed high satisfaction with the procedure (median 5.0 [5.0–5.0]) and reported a strong positive impact on their lives (median 5.0 [3.0, 5.0]). Most patients indicated they would have preferred to undergo the procedure earlier if possible (median 5.0 [5.0–5.0]) and strongly believed that VVS should be offered earlier in the treatment process (median 5.0 [5.0–5.0]). Notably, patients overwhelmingly reported that they would recommend VSS to others with similar conditions (median 5.0 [5.0–5.0]). While some patients found the post-procedural recovery challenging (median 2.0 [1.0–4.0]), the overall sentiment toward the procedure was highly positive.

A logistic regression analysis was performed to identify factors associated with severe MIDAS Grade IV. Table 3 presents the multivariate model results, while univariate models are provided in Supplementary Table S1. The analysis revealed that undergoing VSS was significantly associated with a reduced likelihood of having a severe MIDAS score. Specifically, patients in the VSS group had lower odds of severe disability compared to those in the MT group (odds ratio [OR] = 0.174; 95% confidence interval [CI]: 0.053–0.570; *p* = 0.004). This suggests that VSS is protective against severe headache-related disability. Additionally, the presence of PT in the past three months was a significant predictor of a severe MIDAS score (OR = 1.439; 95% CI: 1.129–1.835; *p* = 0.003), indicating that recent PT contributes to higher disability levels. Other variables, including age at diagnosis, and obesity, did not show a significant association with severe MIDAS scores in the multivariate model (*p* > 0.05) (Table 4).

## 4. Discussion

In this study, we compared disability and functional outcomes between two groups of female patients with IIH who had resolved papilledema: those treated with standard MT and those who underwent VSS. Our findings indicate that patients in the VSS group reported lower disability levels, higher treatment satisfaction, and better overall functional outcomes compared to the MT group.

### 4.1. Baseline Characteristics and Comorbidities

While both groups were matched for age and presence of papilledema, some differences in baseline characteristics were observed. Although the time since diagnosis was shorter in the VSS group compared to the MT group (5.9 ± 6.1 years vs. 8.0 ± 5.4 years, *p* = 0.066), this difference was not statistically significant. Importantly, the majority of VSS patients (91.9%) had been diagnosed for at least two years prior to enrollment, suggesting that both groups consisted of chronic IIH patients.

The MT group had significantly higher rates of polycystic ovary syndrome (PCOS) and fibromyalgia compared to the VSS group (PCOS: 21.9% vs. 2.8%, *p* = 0.010; fibromyalgia: 8.1% vs. 0.0%, *p* = 0.013). Given that both conditions are often underdiagnosed and may be brought to medical attention by patients experiencing persistent symptoms, it is possible that patients in the VSS group, who reported better daily functioning and higher treatment satisfaction, were less likely to report these comorbidities. Alternatively, the longer duration since diagnosis in the MT group may have allowed more time for these comorbidities to be diagnosed. PCOS is known to have a high comorbidity with IIH [25], which may contribute to the observed differences.

A larger proportion of patients in the VSS group reported experiencing PT at diagnosis compared to the MT group (78.4% vs. 55.6%, *p* = 0.019). This may partially explain why patients in the VSS group sought alternative treatments, as MT has limited effectiveness for PT [26,27].

Radiologically, both groups exhibited similar degrees of venous sinus stenosis at diagnosis, with over 90% having a CCS ≤ 5. While the presence of an empty sella turcica was significantly higher in the VSS group (84.6% vs. 52.4%, *p* = 0.004), the overall IIHRS did not differ significantly between groups. These findings align with existing research on radiological features of IIH [22,28,29].

### 4.2. Functional Outcomes and Disability

One of the key findings of our study is that the VSS group demonstrated lower rates of disability, as measured by the MIDAS. A smaller percentage of patients in the VSS group fell into the severe disability category (MIDAS Grade IV) compared to the MT group (24.3% vs. 47.9%, *p* = 0.017). Specifically, the VSS group reported less functional impairment due to headaches affecting work and school activities.

These results suggest that VSS may offer significant benefits in reducing headache-related disability in IIH patients. Previous studies have documented poor quality of life, depression, and cognitive decline in medically treated IIH patients [30,31,32,33]. Our findings support the notion that MT alone may not adequately address the full spectrum of IIH symptoms, particularly chronic headaches.

To our knowledge, this is the first study evaluating MIDAS scores in IIH patients following VSS. Although the MIDAS was originally designed for migraine assessment, we found it to be a practical tool for evaluating headache-related disability in IIH patients, given the lack of specific outcome measures for this condition.

### 4.3. Patient-Reported Outcomes and Satisfaction

Patients in the VSS group reported higher satisfaction with their treatment and better PROMs compared to the MT group. Significant differences were observed in physical and mental well-being, ability to complete tasks, persistence with work or school, and difficulty maintaining social relationships—all favoring the VSS group (*p* < 0.001 for all comparisons). Notably, both groups reported relatively lower scores regarding difficulty maintaining social relationships, indicating that this may be a persistent challenge for IIH patients regardless of treatment modality.

The high satisfaction rates among VSS patients are noteworthy. Most patients expressed that they would recommend VSS to others with similar conditions (91.9%) and wished they had undergone the procedure earlier (89.2%). Despite some patients finding the post-procedural recovery challenging, the overall sentiment toward VSS was highly positive. These findings suggest that VSS may significantly improve patient satisfaction and quality of life in IIH patients who are refractory or intolerant to medical therapy.

### 4.4. Implications for Treatment Guidelines

Our study highlights the potential benefits of VSS as a primary surgical intervention for selected IIH patients. Currently, VSS is often considered a last-resort treatment after failure of MT [34]. However, the significant improvements in disability and patient satisfaction observed in our study suggest that earlier consideration of VSS may be warranted. Given the minimally invasive nature of VSS and its low complication rates compared to other surgical options like V-P shunting or ONSF [35,36,37], it may offer a more favorable risk-benefit profile.

The persistence of functional disability and impaired quality of life in the MT group, despite the resolution of papilledema, indicates that ophthalmological outcomes alone may not fully capture the patient’s experience of IIH. Treatment guidelines should consider incorporating patient-reported outcomes and functional measures to provide a more holistic approach to managing IIH.

### 4.5. Limitations

While our study provides valuable insights, several limitations should be acknowledged. The cross-sectional design limits our ability to establish causality, and the potential for recall bias exists due to the self-reported nature of some outcomes. The unequal follow-up times between groups and the relatively small sample size may also impact the generalizability of our findings. Additionally, although we attempted to match groups based on age and the presence of papilledema, residual confounding variables may still exist.

## 5. Conclusions

In conclusion, our findings suggest that VSS in IIH patients who have failed medical treatment is associated with lower disability levels and higher patient satisfaction compared to standard medical therapy. These results support the notion of considering VSS as a valuable treatment option earlier in the management of selected IIH patients. Future prospective studies with larger cohorts are needed to further elucidate the long-term outcomes, optimal patient selection criteria, and timing for this intervention.

## Figures and Tables

**Table 1:** Demographic, baseline clinical characteristics, symptoms, and radiological findings of study participants.

Category	MT	VSS	*p*-Value
*n* (%)	74 (66.7)	37 (33.3)	
Age, years	32.9 ± 10.4	32.7 ± 10.0	0.912
Time since diagnosis, years	8.0 ± 5.4	5.9 ± 6.1	0.066
Papilledema at diagnosis, *n* (%)	63 (85.1)	28 (75.7)	0.222
Opening pressure at diagnosis, mm H_2_O	377.1 ± 103.4	330.5 ± 103.0	0.044
BMI at diagnosis	30.8 ± 7.4	32.2 ± 6.0	0.338
Any documented computerized Visual field defect at diagnosis, *n* (%)	28 (62.2)	24 (70.6)	0.438
Comorbidities			
Obesity (BMI ≥ 30), *n* (%)	33 (47.1)	24 (66.7)	0.056
Diabetes mellitus, *n* (%)	1 (1.4)	2 (5.6)	0.209
Hypertension, *n* (%)	8 (11.0)	2 (5.6)	0.358
Polycystic ovary syndrome (PCOS), *n* (%)	16 (21.9)	1 (2.8)	0.010
Hypothyroidism, *n* (%)	7 (9.6)	1 (2.8)	0.200
Hypertriglyceridemia, *n* (%)	7 (9.6)	1 (2.8)	0.194
Additional diagnosis of depression or anxiety since IIH was diagnosed, *n* (%)	7 (9.6)	5 (13.5)	0.517
PTSD, *n* (%)	1 (2.7)	0 (0.0)	0.155
Fibromyalgia *n* (%)	3 (8.1)	0 (0.0)	0.013
Pulsatile tinnitus (PT) at diagnosis, *n* (%)	40 (55.6)	29 (78.4)	0.019
Radiological features			
CCS at diagnosis ≤ 5, *n* (%)	67 (90.5)	34 (97.1)	0.217
Empty sella turcica, *n* (%)	33 (52.4)	22 (84.6)	0.004
Optic nerve diameter over 5 mm, *n* (%)	45 (71.4)	18 (69.2)	0.836
Flattened sclera, *n* (%)	30 (47.6)	14 (38.9)	0.400
Radiological score (IIHRS)	2,0 [1.0, 3.0]	2.0 [1.0, 2.0]	0.274

Continuous variables are presented as mean ± SD. Categorical variables are presented as *n* (%). Ordinal variables are presented as median [IQR]. Idiopathic Intracranial Hypertension Radiological Score (IIHRS), post-traumatic stress disorder (PTSD), Combined Conduit Score (CCS), Medical treatment (MT), Venous sinus stenting (VSS), Body index mass (BMI).

**Table 2:** Post-treatment functional outcomes.

Category	MT	VSS	*p*-Value
*n*	93 (69.9)	40 (30.1)	
PT in the past 3 months/post-stenting, *n* (%)	37 (50.7)	6 (16.2)	<0.001
MIDAS grades	3.0 [1.0, 4.0]	1.0 [1.0, 3.5]	0.025
Severe MIDAS (Grade IV), *n* (%)	35 (47.9)	9 (24.3)	0.017
Physical well-being	1.0 [0.0, 4.0]	4.0 [3.0, 5.0]	<0.001
Task completion	1.0 [0.0, 4.5]	4.0 [3.0, 5.0]	<0.001
Work/school persistence	1.0 [0.0, 4.0]	5.0 [3.0, 5.0]	<0.001
Mental well-being	1.0 [0.0, 3.0]	4.0 [3.0, 5.0]	<0.001
Difficulty maintaining social relationships	0.0 [0.0, 1.0]	1.0 [1.0, 5.0]	<0.001
Difficulty remembering where you left things, *n* (%)	22 (32.8)	14 (37.8)	0.608
Difficulty remembering tasks, *n* (%)	25 (36.8)	15 (40.5)	0.703

Continuous variables are presented as mean ± SD. Ordinal variables are presented as median [IQR]. Categorical variables are presented as *n* (%); MIDAS grades are a validated ordinal variable ranging from 1 to 4 and presented as median and IQR. It is based on the continuous MIDAS score variable, presented as the mean and SD of the sum of days. The variables, namely ‘physically feels better post-treatment’, ‘finishes tasks better post-treatment’, ‘persistent at work or school post-treatment’, ‘feels happier post-treatment’, and ‘disconnected relationships post-treatment’, are self-reported ordinal questions, each rated on a scale from 0 to 5. The variables ‘difficulty remembering where you left things’, ‘maintaining social relationships’, and ‘remembering tasks’ are self-reported yes/no questions and are presented as categorical variables, *n* (%).

**Table 3:** Procedural outcomes in the VSS group.

Variable		Result
Time since VSS months	Mean ± SD	22.4 ± 16.8
Self-reported ordinal questions (0–7)		
Extent of headache discomfort before VSS	Median [IQR]	7.0 [6.0, 7.0]
Extent of headache discomfort currently		3.0 [0.0, 4.0]
Self-reported ordinal questions (1–5)		
The VSS had a positive impact on my life	Median [IQR]	5.0 [3.0, 5.0]
Satisfaction with the VSS procedure		5.0 [5.0, 5.0]
Post-VSS recovery was difficult		2.0 [1.0, 4.0]
Would prefer to have undergone VSS, if feasible		5.0 [5.0, 5.0]
VSS should have been offered to me earlier		5.0 [5.0, 5.0]
The option of VSS was not presented to me, possibly due to limited awareness among doctors.		5.0 [1.0, 5.0]
Would recommend VSS to patients in my condition		5.0 [5.0, 5.0]

**Table 4:** Predictors of severe MIDA scores.

Variable	*p*-Value	OR	CI
VSS	**0.004**	0.174	0.053–0.569
Current age	0.363	0.977	0.929–1.027
PT in the past 3 months	**0.003**	1.439	1.129–1.833
Obesity (BMI ≥ 30)	0.393	1.551	0.567–4.246

In total, 104 cases were included in the analysis.

## Data Availability

There are no additional data deposited on any other site other than in this manuscript.

## Supplementary Materials

The following supporting information can be downloaded at: https://www.mdpi.com/article/10.3390/diagnostics14222572/s1, Table S1: Univariate models for severe MIDAS Grade 4; Demographic Questionnaire.
