# Supplementary material for: Disability and Patient-Reported Satisfaction in Women with Idiopathic Intracranial Hypertension: A Comparative Study of Venous Sinus Stenting and Medical Management
Source: Diagnostics (Basel). 2024 Nov 15;14(22):2572. doi: 10.3390/diagnostics14222572 (PMC11593176; doi:10.3390/diagnostics14222572)
Supplement: Supplementary file 1 [file diagnostics-14-02572-s001.zip › diagnostics-3286009-supplementary.pdf]

Study Questionnaire

Hello, I'm talking to __________. My name is __________, I am a general physician, and I am doing a study on IIH. This study should help patients in your situation. We would like to ask you to answer a short questionnaire for a few minutes that includes questions about the symptoms you are experiencing and your health status.

The confidentiality of your personal information is guaranteed. In addition to the questionnaire, computerized information will be collected later from the medical records existing in the hospital's system. There is of course no obligation to participate in the study.

Agree / Disagree

If you do not agree to enter the medical record, please indicate this.

We thank you again for your investment and patience on behalf of the research team.

First name and last name:

Questionnaire filling date:

Demographic questionnaire

1. ID number: ________________

2. Surname: ______________ First name: __________________

3. Date of birth: _____________

4. Country of birth: _______________ Year of immigration: _________________

5. Gender: male \female

6. Marital status: Married \ Separated \ Divorced \ Widower \ Single

7. Number of children: ______ number of births____

8. Religion: _______________

Weight at Diagnosis: ____________

Height: ____________

IIH diagnosis date:____________

Have you had a stroke? Yes/ No

Opening pressure at the time of diagnosis_____________

Presence of papilledema at diagnosis: Yes/ No

1. Are you currently taking medication for IIH? Yes/No
2. not currently, what medication have you received in the past?
   - o Topamax
   - o Ormox
   - o Both
   - o Other
3. Did you perform an intervention other than medication?
   - o Yes, venous catheterization.
   - o Yes, Shunt
   - o Yes, bariatric surgery.

4. Have you been monitored in the last year?
   - o In the last six months
   - o In the last year
   - o In the last two years
   - o Not tracked
5. If the treatment was stopped, why?
   - o Side Effects
   - o The doctor was impressed you recovered
   - o On your own
   - o Other
6. Are you careful / have you been careful in the past (if not relevant at the moment) about the drug treatment?
   - o I made sure.
   - o I was careful with the most part
   - o I took when I remembered
   - o I didn't care at all.

Please provide details about the medications you take regularly:

**Name of the drug**	**Number of times per day**	**Dose**

For each medication, please write down the number of times a month you take the medication:

**Name of the drug**	**Number of times per month**	**Dose**
Any painkillers		
Paracetamol/ Opthalgin/ Advil/ Nurofen		
Roxet Plus/ Paracetamol Focus		
Rizlat/ Rilert/ Zomig		
Tramdex/Zaldiar/Percocet		
Other__________________		

In the following table, please detail your habits:

**Habits**	**Yes**	**No**	**Per day**
Smoking			
Drug abuse			
Alcohol abuse			

In the following table, if you do not suffer from diseases or other medical problems besides the diagnosis, please mark "No", if yes, please mark "Yes" and specify the year of diagnosis

**Other illness**	**Yes**	**No**	**Year of diagnosis**
Diabetes mellitus			
HTN			
PCO			
Hypothyroidism			
Hypertriglyceridemia			
Anemia			
ADHD			
Psychiatric illness			

Please circle the extent to which you have suffered in the last three months from "tinnitus" (ringing in the ears), where "0" is not at all and "7" is non-stop:

0	1	2	3	4	5	6	7

Please circle the extent to which you suffered, upon diagnosis, from "tinnitus" (ringing in the ears), where "0" is not at all and "7" is non-stop:

0	1	2	3	4	5	6	7

HIT-6™ Headache Impact Test

Migraine Disability Assessment Test (MIDAS):

Answer the following questions about the headaches of any kind you've experienced over the past three months. Use zero for questions where you have not experienced any activity disruption during the past three months.

1. How many days have you missed work or school because of a headache? _______

2. Not including the days from question one, how many days have you lost productivity by at least half at school or work? _______

3. How many days have you skipped performing household chores or regular household activities because of a headache? _______

4. Not including the days from question two, how many days was your productivity in performing household chores reduced by at least half? _______

5. How many days did you miss leisure or social activities because of your headaches?

- How many days have you had a headache? Note that if a headache lasted more than one day, count each day.
- On average, how painful were the headaches? Use a scale of 0-10 with 0 being no pain and 10 being the most painful.

Please mark how much you agree with the following statements, the statements refer to your situation in the last three months, under the drug treatment:

	**I don't agree at all**	**Partially incorrect**	**Moderately agree**	**Partially agree**	**Strongly Agree**	**Irrelevant**
I feel an improvement in my general physical condition	1	2	3	4	5	-
I can finish tasks better	1	2	3	4	5	-
I manage to keep up with my work\ keep up with my studies	1	2	3	4	5	-
I feel happier	1	2	3	4	5	-
I cut ties with people	1	2	3	4	5	-
I felt a decrease in the frequency of headaches	1	2	3	4	5	-
I feel less ringing in my ears (tinnitus) I feel less ringing in my ears (tinnitus)	1	2	3	4	5	-
I feel discouraged by the ability of medicine to treat my illness	1	2	3	4	5	-
In general, I am satisfied with the medication	1	2	3	4	5	-

Please circle the appropriate answer regarding your feelings, in the last three months, under medication:

	**YES**	**NO**
A tingling sensation in the palms of the hands	1	2
A tingling sensation in the feet	1	2
Bitter taste in the mouth	1	2
general weakness	1	2
Pain in the waist	1	2

To what extent do you suffer from side effects of the drug treatment with Ormox/Topamax when "0" is not at all and "7" it bothers me a lot:

0	1	2	3	4	5	6	7

Please circle your feeling regarding the difficulty in daily activities, with the diagnosis of the disease and also, in the last three months under the treatment:

	**Under treatment**	**At diagnosis**
Difficulty watching TV for a long time	YES\ NO	YES\ NO
Difficulty in prolonged reading	YES\ NO	YES\ NO
Difficulty driving	YES\ NO	YES\ NO
Difficulty learning new information	YES\ NO	YES\ NO
Difficulty remembering where I put things	ES\ NO	YES\ NO
Difficulty remembering whether I have completed tasks	YES\ NO	YES\ NO

Please circle the appropriate answer for the following questions

Since starting the medication, have you experienced weight loss? Yes \ No

Since the beginning of the drug treatment, have you experienced weight gain? Yes \ No

If there is a change in your weight, please answer the following sections:

Please circle the most appropriate answer for you:

	I don't agree at all	Partially disagree	Moderately agree	Partially agree	Strongly Agree
Since starting the medication, I have changed my eating habits	1	2	3	4	5
Since starting the medication, I have changed my exercise habits	1	2	3	4	5

Fill in the following table:

Your weight at the time of diagnosis:	
Your weight now:	

Supplemental table S1: Univariate models for severe MIDAS (grade 4)

Variable	P-val	OR
**Catheterized**	**0.019**	**0.349**
**Age**	0.912	1.002
**Time since diagnosis**	0.873	1.006
**Uramox/Topamax**	0.311	1.486
**Depression Medication**	0.901	1.081
**PT at diagnosis**	0.689	0.974
**PT in the past 3 months**	**0.004**	**1.326**
**CCS**	0.991	0.999
**CCS≤5**	0.737	1.239
**BMI**	0.132	1.045
**Morbid obesity**	0.199	1.828
**Obesity**	0.239	1.611
**Overweight**	0.647	1.309
**Weight at diagnosis**	0.473	1.007
**Weight loss since diagnosis**	0.723	1.155
**Height**	0.493	9.175
**Categorized height**	0.732	1.103
**Papilledema**	0.086	0.414
**Opening pressure at diagnosis**	0.832	1.000
